# Impact of Antimicrobial Mouthwash on Outcomes of Er: YAG Laser Versus Scalpel Frenectomy: A Retrospective Longitudinal Cohort Study

**DOI:** 10.3390/jcm15062419

**Published:** 2026-03-21

**Authors:** Seval Ceylan Şen, Özlem Saraç Atagün, Gülbahar Ustaoğlu, Şeyma Çardakcı Bahar, Zeynep Hazan Yıldız, Burak Çevik

**Affiliations:** Department of Periodontology, Gulhane Faculty of Dentistry, University of Health Sciences, Ankara 06010, Turkey; ozlem.saracatagun@sbu.edu.tr (Ö.S.A.); gulbahar.ustaoglu@sbu.edu.tr (G.U.); seyma.cardakcibahar@sbu.edu.tr (Ş.Ç.B.); zeynephazan.yildiz@sbu.edu.tr (Z.H.Y.); burak.cevik@sbu.edu.tr (B.Ç.)

**Keywords:** Er: YAG laser, frenectomy, conventional scalpel surgery, antimicrobial agents, wound healing, patient-reported outcomes

## Abstract

**Objective**: This study compared the clinical and patient-reported outcomes of Er: YAG laser-assisted versus conventional scalpel frenectomy, while evaluating the adjunctive impact of postoperative antimicrobial mouthwashes on wound healing and periodontal parameters. **Methods**: A total of 102 patients who underwent labial frenectomy were included in this retrospective longitudinal cohort study. Participants were allocated into four groups based on the surgical approach (Er: YAG laser or conventional scalpel) and the postoperative mouthwash protocol (Kloroben^®^ or Klorhex Plus^®^). Clinical assessments were performed at baseline and at 7, 14, and 28 days postoperatively. Wound healing, evaluated using the Wound Healing Index, was defined as the primary outcome. Secondary outcomes included periodontal clinical parameters, epithelialization status, postoperative pain, bleeding, and analgesic consumption. To control potential confounders, multivariable regression analysis was performed alongside standard parametric and nonparametric tests, with *p* < 0.05 considered statistically significant. **Results**: All treatment protocols resulted in significant improvements over time (*p* < 0.001). However, Er: YAG laser–assisted frenectomy was associated with significantly better periodontal indices, superior wound-healing scores, and more favorable patient-reported outcomes than the conventional scalpel technique at all postoperative evaluations (*p* < 0.001). On day 7, ‘Very Good’ healing was observed in 70.2% of the laser groups, compared with 14.4% in the CS groups (*p* = 0.001). Group 4 showed the lowest mean VAS scores (0.04 ± 0.20) and the lowest analgesic consumption by day 7. Multivariable analysis confirmed that the surgical technique was the strongest independent predictor of superior wound healing (*p* < 0.05), regardless of age, gender, smoking, or systemic disease. Notably, frenulum type was not significantly associated with wound healing or pain outcomes (*p* > 0.05). **Conclusions**: Within the limitations of this study, Er: YAG laser-assisted frenectomy was observed to provide favorable wound healing outcomes compared to the conventional technique. Furthermore, our findings show that anatomical variations in frenulum type do not significantly influence the quality or speed of recovery. These findings suggest that the choice of surgical modality and postoperative chemical support are more critical determinants of early clinical success than the anatomical variations of the frenulum.

## 1. Introduction

The frenulum is a mucosal fold, usually containing closed muscle fibers, that connects the lips and cheeks to the alveolar mucosa and/or gingiva and underlying periosteum. The frenulum problem is most common on the labial surface between the maxillary and mandibular central incisors and in the canine and premolar areas [1].

The labial frenulum is a mucosal fold that stabilizes lip movement; however, aberrant attachments (papillary or papillary penetrating) are often associated with gingival recession, midline diastema, and impaired oral hygiene [1,2,3]. While conventional scalpel (CS) frenectomy remains a routine procedure, it often results in significant surgical trauma, the necessity for sutures, and healing by secondary intention, leading to increased postoperative discomfort [4,5].

Previous research has extensively documented the challenges associated with conventional scalpel surgery, primarily focusing on intraoperative bleeding, the necessity for sutures, and significant postoperative edema [6,7]. While various laser systems, including diode and CO_2_, have been introduced to mitigate these issues, each carries specific thermal characteristics that may influence the biological response of the oral mucosa [8]. Recent advancements focus on minimizing surgical morbidity through laser technology. Among various systems, the Erbium:yttrium-aluminum-garnet (Er: YAG) laser (2940 nm) offers distinct advantages over “hard” lasers such as CO_2_ or diode lasers. Its wavelength coincides with the maximum absorption peak of water, enabling “cold ablation.” [7,9]. While diode lasers (e.g., 940–980 nm) primarily utilize photothermal effects that can lead to deep carbonization and subsequent delayed secondary intention healing, the 2940 nm wavelength of the Er: YAG laser targets water molecules directly, preventing the ‘charring’ effect often seen with CO_2_ or diode systems [10]. This distinction is critical at the cellular level; minimizing thermal stress preserves the viability of the adjacent periodontal ligament and periosteum, thereby facilitating more rapid fibroblast migration and re-epithelialization during the early stages of recovery. Recent studies by Sayed Taha et al. [7] and Sarmadi et al. [11] have highlighted the efficiency of Er: YAG lasers in promoting faster tissue repair, yet the variability in healing outcomes suggests that surgical technique alone may not be the sole determinant of recovery quality. Unlike diode lasers, which primarily rely on thermal energy and can cause deep carbonization, the Er: YAG laser enables precise tissue removal with minimal collateral thermal damage, thereby reducing the risk of delayed healing and postoperative pain [8]. Despite the higher equipment cost, the clinical rationale for the use of Er: YAG lies in superior patient comfort, reduced chairside time, and potentially faster recovery, which may offset the initial investment [9,12].

Despite the documented benefits of laser technology, there is a profound lack of consensus regarding the optimized postoperative management of the surgical site. Specifically, the synergistic impact of combining advanced ‘cold ablation’ techniques with targeted antimicrobial and anti-inflammatory chemical adjuncts remains poorly understood. Most existing literature focuses on either the surgical tool or the topical agent in isolation, leaving a gap in clinical knowledge regarding how these variables interact to influence long-term periodontal stability and patient-reported morbidity [12,13]. While Chlorhexidine (CHX) is the gold standard for anti-plaque control, the integration of anti-inflammatory agents, such as Flurbiprofen, into mouthwashes (e.g., Klorhex Plus^®^) may synergistically enhance the healing environment [13,14]. The strategic integration of Flurbiprofen into a Chlorhexidine-based mouthwash (e.g., Klorhex Plus^®^) aims to create a localized dual-action reservoir at the surgical site. By directly targeting the inflammatory cascade at the mucosal interface, this synergistic approach is designed to attenuate the early post-surgical pain response, potentially reducing the patient’s reliance on systemic analgesia and minimizing the overall pharmacological burden during the critical first 7 days [13,14].

There is limited consensus on whether these adjuvant methods significantly differentiate the recovery profiles of laser-assisted versus conventional techniques [15,16]. Furthermore, while the anatomical classification of the labial frenulum is well-established regarding its impact on orthodontics and periodontics, there is a significant lack of clinical evidence concerning whether different frenulum attachments (e.g., gingival vs. papillary penetrating) demand distinct surgical approaches or exhibit varied healing kinetics [17,18]. This study seeks to resolve this clinical ambiguity by determining if anatomical complexity dictates the recovery profile or if the choice of surgical modality remains the primary determinant of success.

The rationale for the current study lies in the necessity to establish a more comprehensive clinical protocol for labial frenectomy. By evaluating the Er: YAG laser’s precise tissue management alongside potent flurbiprofen-containing adjuncts, this research seeks to determine whether a combined approach can significantly reduce the pharmacological burden on the patient while accelerating the return to functional normalcy. This evidence is crucial for refining clinical decision-making in periodontal plastic surgery.

The specific objectives of this study are

1. To compare the clinical wound-healing efficiency of Er: YAG laser versus conventional scalpel surgery.
2. To evaluate the adjunctive impact of different antimicrobial mouthwash protocols (Kloroben^®^ vs. Klorhex Plus^®^) on postoperative pain, bleeding, and analgesic consumption.
3. To assess whether the anatomical type of the frenulum significantly influences the recovery profile across different treatment modalities.

## 2. Materials and Methods

This retrospective, longitudinal cohort clinical study was based on the records of patients who underwent labial frenectomy at the Department of Periodontology, Gulhane Faculty of Dentistry, between January 2023 and March 2025. Among 463 patients who presented with indications for frenectomy during this period, a total of 102 patients treated using either the conventional surgical technique or the Er: YAG laser were included in the study.

The data were collected retrospectively from records obtained for routine clinical purposes, and the study was conducted in accordance with the principles of the Declaration of Helsinki. At the time of their initial surgical procedures, all patients provided written informed consent, which included permission for their clinical data and records to be used for future scientific research and educational purposes. The institutional ethics committee explicitly reviewed the study protocol for the retrospective analysis of these pre-existing records and waived the requirement for a new, study-specific informed consent in accordance with the prior authorizations obtained. Ethical approval was obtained from the Gulhane Training and Research Hospital Ethics Committee, Ankara, Türkiye (Approval No: 2025/264, Date: 6 May 2025), and the study was registered at ClinicalTrials.gov (Identifier: NCT07054021). This study was reported in accordance with the Strengthening the Reporting of Observational Studies in Epidemiology (STROBE) statement (Supplementary Materials).

### 2.1. Sample Size Calculation

A post hoc power analysis was performed using G*Power (version 3.1.9.7) to assess the adequacy of the sample size in this retrospective, clinical, comparative longitudinal cohort study. The calculation was based on the primary outcome, wound healing. The final analysis included 102 patients distributed across four groups: Group 1 (n = 27), Group 2 (n = 24), Group 3 (n = 25), and Group 4 (n = 26). Assuming an F-test experimental design, a large effect size (f = 0.40) and a Type I error rate (α) of 0.05, the achieved post hoc statistical power was calculated to be 93%, indicating that the sample size was statistically sufficient to detect meaningful intergroup differences [11].

### 2.2. Study Population, Inclusion, and Exclusion Criteria

Based on the clinical records, participants were distributed into four study groups according to the surgical intervention performed and the postoperative mouthwash protocol prescribed:

- Group 1 (CS Technique + Kloroben^®^ Mouthwash): Frenectomy performed using the conventional scalpel technique followed by a 7-day postoperative Kloroben^®^ mouthwash (n = 27).
- Group 2 (Er: YAG laser + Kloroben^®^ Mouthwash): Frenectomy performed using Er: YAG laser surgery followed by a 7-day postoperative Kloroben^®^ mouthwash use (n = 24).
- Group 3 (CS Technique + Klorhex Plus^®^ Mouthwash): Frenectomy performed using the conventional scalpel technique followed by a 7-day postoperative Klorhex^®^ Plus mouthwash (n = 25).
- Group 4 (Er: YAG laser + Klorhex Plus^®^ Mouthwash): Frenectomy performed using Er: YAG laser surgery followed by a 7-day postoperative Klorhex Plus^®^ mouthwash use (n = 26).

The inclusion criteria for participant selection were as follows: individuals who were systemically healthy or had systemic conditions that do not impair wound healing; aged between 18 and 65 years; presenting with maxillary central and lateral incisors and canines; who had completed initial periodontal therapy prior to frenectomy; exhibited full-mouth plaque and bleeding scores ≤ 20%; had no history of connective tissue disorders; and had not undergone any restorative or prosthodontic treatment or periodontal surgical procedures that could potentially alter the anatomy of the relevant region.

The exclusion criteria were defined as follows: individuals receiving medications known to impair wound healing or affect gingival tissues; heavy smokers; patients with systemic diseases known to compromise wound healing; individuals diagnosed with connective tissue disorders; cases with incomplete clinical or medical records; and patients who did not complete a minimum follow-up period of at least 4 weeks.

### 2.3. Surgical Procedures

Patients underwent an intraoral examination before the procedure and were classified as mucosal, gingival, papillary, or papillary penetrating based on examination of the frenulum. Their age, gender, smoking status, and systemic diseases were recorded in writing.

### 2.4. Conventional Scalpel Technique Surgery

The frenectomy site was gently anesthetized using articaine hydrochloride 80 mg/2 mL + epinephrine bitartrate 0.02 mg/2 mL (Maxicaine Fort^®^, Vem Ilac, Istanbul, Turkey).

**Figure 1:**
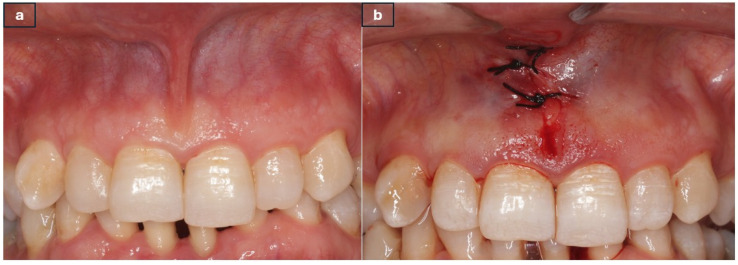
Conventional Scalpel Surgery (**a**) Preoperative appearance (**b**) Postoperative appearance.

In patients undergoing traditional techniques, the upper lip was extended, and a straight homeostat was attached to the frenulum at the depth of the vestibular fold. Triangular incisions were made using a 15C scalpel (HM0240^®^, Beybi, Ümraniye, Istanbul, Turkey) until the labial frenulum was separated from the soft tissue. After excision of the frenulum, muscle fibre dissection was performed with curved forceps to separate the lateral walls from the submucosa and periosteum. A 4/0 silk suture was used for primary wound closure (DOGSAN^®^, Beşiktaş, Istanbul, Turkey) (Figure 1a,b).

### 2.5. Er: YAG Laser Surgery

Local anesthesia was achieved by gentle infiltration at the frenectomy site using articaine hydrochloride 80 mg/2 mL combined with epinephrine bitartrate 0.02 mg/2 mL (Maxicaine Fort^®^, Vem İlaç, Istanbul, Turkey).

**Figure 2:**
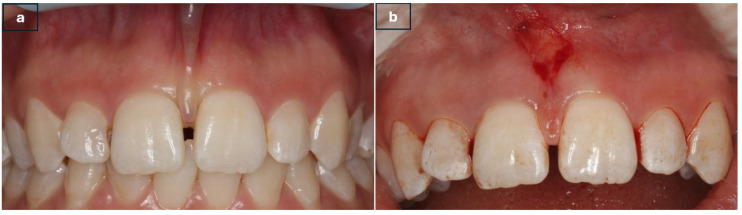
Er: YAG Surgery (**a**) Preoperative appearance (**b**) Postoperative appearance.

In cases where laser technology was applied, an Er: YAG laser system (AT Fidelis Plus III, Fotona^®^, Ljubljana, Slovenia) operating at 2940 nm was used with an R014 handpiece and a cylindrical sapphire fiber tip measuring 8/1.3 mm. The laser parameters were set to very long pulse (VLP) mode with a pulse duration of 1000 μs, a pulse energy of 150 mJ, and a pulse frequency of 10 Hz. All laser settings were selected in accordance with the manufacturer’s recommendations for frenectomy procedures (Figure 2a,b).

### 2.6. Postoperative Care

Patients were provided with written information on postoperative oral care. Patients were advised to be cautious and avoid mechanical trauma, flossing, and chewing. Gentle tooth brushing was permitted using a surgical toothbrush (Surgical Mega Soft, Curaprox^®^, Kriens, Switzerland). In the CS technique, sutures placed in the surgical area were removed one week (7 days) after the procedure.

1st and 2nd test groups were instructed to gently rinse the wound area twice daily for 7 days with Kloroben^®^ mouthwash (Drogsan, Ankara, Turkey) containing 120 mg (0.12%) chlorhexidine gluconate and 150 mg (0.15%) benzidamine hydrochloride; 3rd and 4th test groups were also advised to gently rinse the wound area twice daily for 7 days with Klorhex Plus^®^ mouthwash (Drogsan, Ankara, Turkey), which contains 200 mL of mouthwash, 0.5 g of flurbiprofen, and 0.24 g of chlorhexidine digluconate. Only 500 mg of paracetamol-containing analgesics (Parol^®^ 500 mg, Atabay, Istanbul, Turkey) were prescribed after operations. Patients were advised to use mouthwash until the sutures were removed and asked to record the number of analgesics used and any bleeding during the first week after the operation.

### 2.7. Postoperative Measurements

#### 2.7.1. Clinician-Performed Measurements and Evaluations

All pre- and postoperative clinical measurements and recovery data were recorded. Clinical measurements were recorded in the region of teeth numbered 13 to 23 at baseline (T0), and on the 7th (T1), 14th (T2), and 28th (T3) days following the surgical procedure. The parameters assessed included the Gingival Index (GI) according to Loe and Silness [19], the Plaque Index (PI) based on the Turesky, Gilmore, and Glickman modification of the Quigley-Hein index [20], the papillary bleeding index (PBI) [21], and Probing Depth (PD) [22]. Landry’s wound healing was scored according to WHI [23]. The WHI was recorded on the 7th, 14th, and 28th days. The primary outcome of the study was to measure the status of labial wound healing using WHI, which grades healing on a scale. This index, which ranges from 1 (very poor) to 5 (excellent), evaluates tissue color, response to palpation, presence of granulation tissue, epithelialization of incision margins, and amount of suppuration.

Epithelialization of the surgical region was assessed by observing the application of 3% hydrogen peroxide and whether foaming occurred, and was calculated as a percentage (H_2_O_2_ bubbling) [24]. Complete Epithelization (CE, bubbling) was recorded on the 7th, 14th, and 28th days. CE was evaluated clinically by monitoring the surface characteristics and clarity of the wound contour and recorded as “yes” or “no” [25].

#### 2.7.2. Patient-Recorded Data

The secondary results of the study showed that patients’ postoperative pain was rated on a Visual Analog Scale (VAS) from 0 (no pain) to 10 (the most severe pain imaginable) between days 1 and 7 and at the 2nd post-surgery hour [26]. Additionally, they were asked to document postoperative bleeding and the number of analgesics taken during this period, ensuring that all data were recorded accurately and completely in writing.

**Figure 3:**
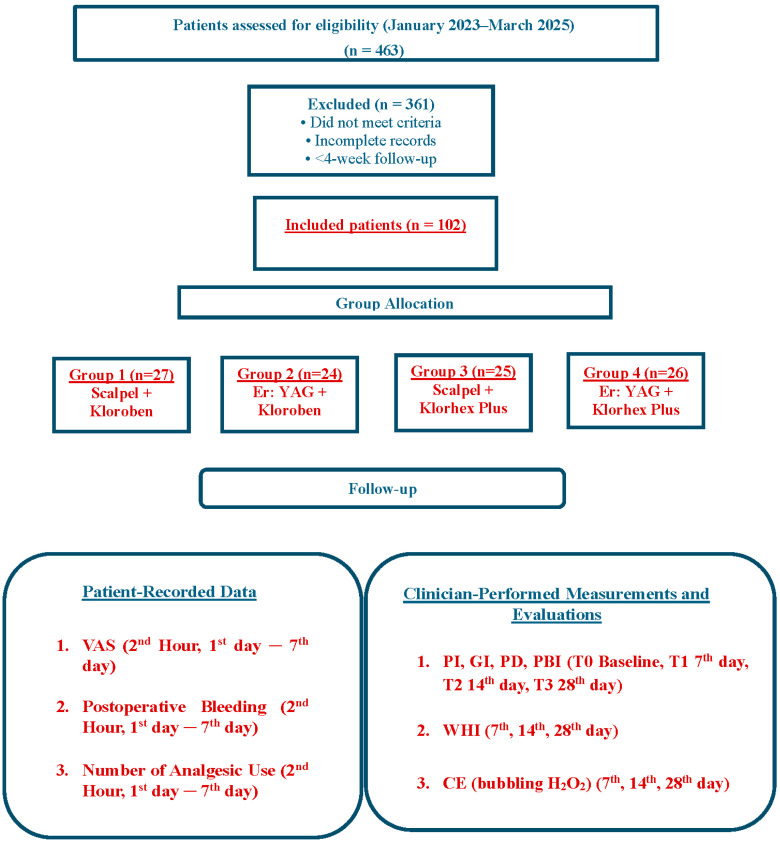
Flow diagram of patient selection, group allocation, and follow-up.

Generative AI was utilized solely for auxiliary text processing and linguistic refinement during the preparation of this study, with the authors maintaining full responsibility for the scientific content and interpretations (Figure 3).

### 2.8. Statistical Analysis

The Shapiro–Wilk and Skewness-Kurtosis tests were used to determine whether the continuous measurements in the study were normally distributed. Since these measurements were normally distributed, parametric tests were applied. Descriptive statistics for the study variables were reported as mean, standard deviation, number (n), and percentage (%). “One-way Analysis of Variance (ANOVA)” was used to compare continuous measurements between groups. Following the variance analysis, the “Duncan test” was used to identify different groups. A repeated-measures ANOVA was used to compare measurement periods (times) within each group, and, following this test, the “Bonferroni-corrected post hoc multiple comparison test” was used to identify the measurement times that produced the difference. To ensure the robustness of the results, ordinal data (WHI and analgesic consumption) were also verified using the nonparametric Kruskal–Wallis test. The results were consistent with the parametric ANOVA outputs, justifying the reported analysis. The “Chi-square or Fisher’s Exact” tests were calculated to determine the relationships between categorical variables. Pearson correlation coefficients were calculated to determine the relationships between continuous measurements. To address potential selection bias and the observed age imbalance between groups (*p* = 0.024), a multivariable logistic regression analysis was conducted using the ‘Enter’ method. The primary dependent variable was ‘wound healing,’ defined as achieving an ‘Excellent’ score on the Landry Wound Healing Index (WHI) by the end of the follow-up period. Age, gender, smoking status, and systemic disease were included in the model as covariates to calculate adjusted Odds Ratios (OR) and 95% Confidence Intervals (CI). This modeling approach allowed the isolation of the independent effect of surgical intervention while controlling demographic variation. Prior to analyzing ordinal data, the assumption of normality was checked using the Shapiro–Wilk test; in addition to parametric tests (ANOVA), the robustness of the results was confirmed using non-parametric tests. The statistical significance level was set at *p* < 0.05, and the SPSS (IBM SPSS for Windows, ver. 27) statistical package was used for the analysis.

## 3. Results

### 3.1. Demographic and Clinical Characteristics

Groups were homogenous regarding gender, toothbrushing frequency, smoking, systemic disease, and frenulum type (*p* > 0.05). The only significant difference was in age distribution (*p* = 0.024); the 18–24 age group was predominantly in Groups 3 (32.4%) and 4 (37.8%), while the 25–44 age group was concentrated in Groups 1 (31.0%) and 2 (35.7%). Although systemic disease prevalence varied numerically (absent in Group 2 vs. 55.6% in Group 4), this difference remained statistically non-significant (*p* = 0.071) (Supplementary Table S1).

Table 1 shows the overall distribution of demographic and clinical characteristics for the 102 patients included in the study and the four treatment groups. When the general population is examined, the gender distribution of patients is balanced (48% male, 52% female). The age distribution is similarly spread across all three groups, with the largest group being in the 18–24 age range (36.3%). Most patients brush their teeth twice a day (82.4%) and do not smoke (79.4%). The presence of systemic disease is generally low (8.8%). In terms of frenulum type distribution, the most common type is the “Papillary” frenulum (46.1%).

### 3.2. Clinician-Performed Measurements and Evaluations

#### 3.2.1. Periodontal Clinic Parameters

Table 2 presents the comparative analysis of clinical parameters (PI, PD, GI, and PBI) across the four treatment groups. At baseline, all groups were homogeneous (*p* > 0.05$). However, statistically significant intergroup differences were observed at all post-treatment intervals (days 7, 14, and 28) for all clinical parameters (*p* = 0.001). Group 4 consistently demonstrated the lowest mean values, indicating the greatest clinical improvement, while Group 1 generally showed the least improvement. Intragroup analysis revealed that all parameters significantly decreased from baseline to day 28 across all protocols (*p* = 0.001), confirming their overall effectiveness. Notably, PI values initially increased on day 7, then fell significantly below baseline levels by days 14 and 28 in all groups.

#### 3.2.2. Wound Healing Parameters

Table 3 presents the distribution of Landry scores across the four treatment groups at 7, 14, and 28 days. Statistically significant differences were observed at all time points (*p* = 0.001), suggesting a positive association between laser-assisted techniques and the quality and speed of healing. On day 7, ‘Very Good’ recovery was predominantly observed in the laser groups (70.2% in Groups 2 and 4), whereas ‘Poor’ and ‘Good’ scores were concentrated in the conventional groups. By day 14, the ‘Excellent’ category was dominated by laser groups (73.9%), whereas only conventional groups remained in the ‘Good’ category. By day 28, although most patients reached ‘Excellent’ status, all remaining ‘Very Good’ cases were in the conventional groups (Groups 1 and 3), with Group 4 showing the highest overall ‘Excellent’ recovery rate (28.6%).

#### 3.2.3. Complete Epithelization Parameters

Table 4 presents the distribution of Complete Epithelialization (CE), assessed via the H_2_O_2_ bubbling test, across the four groups at 7, 14, and 28 days. A highly significant intergroup difference was observed on day 7 (*p* = 0.001), with most patients without bubbling (indicating advanced healing) in the laser groups (76.9% of the total non-bubbling cases; Group 2: 35.9%; Group 4: 41.0%). In contrast, all patients in Group 1 (100%) showed bubbling. By day 14, although the proportion of patients without bubbling increased in all groups, the difference did not reach statistical significance (*p* = 0.063), with bubbling absent in the laser groups. By day 28, intergroup differences were non-significant (*p* = 0.579) as epithelialization was nearly complete across all protocols; the few remaining cases with incomplete epithelialization were limited to conventional groups (Groups 1 and 3).

#### 3.2.4. Multivariable Regression Analysis of Wound Healing (Adjusted for Age, Gender, Smoking Status, and Systemic Disease)

To evaluate the potential impact of baseline characteristics on wound healing and to minimize allocation bias, a multivariable regression analysis was performed. In this model, ‘wound healing’ was defined as the dependent variable, while age, gender, presence of systemic disease, and smoking status were included as independent variables (covariates). The results of this analysis (Table 5) indicated that age, gender, systemic disease, and smoking were not dominant confounders explaining the fundamental differences between the groups. These findings suggest that the statistical homogeneity between the groups was maintained and that the observed clinical outcomes were independently associated with the treatment interventions.

Specifically, the multivariable model demonstrated that the age distribution of the patients did not significantly influence the probability of achieving superior wound healing (OR: 0.967, 95% CI: 0.915–1.022, *p* = 0.236). Similarly, gender, smoking, and systemic disease were not found to be significant independent predictors (*p* > 0.05). These findings support the observed clinical advantages of the Er: YAG laser and flurbiprofen-containing mouthwash protocols, which remain statistically robust even after adjusting for baseline demographic imbalances.

### 3.3. Patient-Recorded Data

#### 3.3.1. VAS and Analgesic Use

Table 6 compares the postoperative pain experience (VAS) and analgesic requirements across the four treatment groups over a 7-day period. VAS scores decreased significantly in all groups over time (*p* = 0.001). While no significant difference in pain intensity was observed at 2 h post-treatment (*p* = 0.141), laser groups reported significantly lower pain from day 1 onward, particularly on days 5, 6, and 7, during which Group 4 achieved nearly pain-free levels (VAS: 0.04). Intergroup differences in analgesic consumption were even more pronounced; Group 1 required significantly more medication within the first 2 h (1.22 units, *p* = 0.001) and on day 1 (0.74 tablets, *p* = 0.009) compared to laser groups. Although analgesic needs decreased rapidly across all protocols after day 2 (*p* = 0.001), laser-assisted treatments consistently required less medication in the early postoperative phase.

#### 3.3.2. Postoperative Bleeding

Table 7 presents the temporal distribution of postoperative bleeding across the four treatment groups. Laser-assisted groups (Groups 2 and 4) showed a significant advantage in bleeding control, particularly in the early postoperative phase. Within the first two hours. A significant intergroup difference was observed (*p* = 0.004): 73.7% of patients with bleeding belonged to the conventional groups (Groups 1 and 3). While the laser groups accounted for 62.6% of those without bleeding. A similar trend persisted on day 2 (*p* = 0.044), with bleeding absent in Group 2 and limited to the non-laser groups. From day 3 onward. Bleeding incidence decreased rapidly across all protocols, with intergroup differences no longer significant. By day 5, no bleeding was reported in any group.

#### 3.3.3. Frenulum Type; Wound Healing and VAS

Frenulum type had no statistically significant impact on Landry healing scores at 7, 14, or 28 days (*p* = 0.349, *p* = 0.818, *p* = 0.126, respectively). By day 14, healing was mostly ‘Good’ or ‘Very Good’, with ‘Poor’ scores limited to Gingival and Papillary types. All Mucosal and Papillary Penetrating types achieved ‘Very Good’ or ‘Excellent’ status, while others followed closely. By day 28. The vast majority (83–100%) reached ‘Excellent’ healing, notably. Mucosal and Papillary Penetrating types showed 100% ‘Excellent’ recovery, whereas a small percentage of Gingival (12.5%) and Papillary (17.0%) types remained at ‘Very Good’ (Supplementary Table S2).

VAS scores significantly decreased over time across all frenulum types (*p* = 0.001 for each), indicating a normal postoperative healing process. However, inter-group comparisons revealed no statistically significant differences at any time point (*p* > 0.05), suggesting that frenulum type does not influence postoperative pain severity (Supplementary Table S3).

## 4. Discussion

The present study aimed to evaluate and compare the clinical outcomes of conventional scalpel-assisted and Er: YAG laser–assisted labial frenectomies across different frenulum types, using various chemical adjuncts. The biological superiority of the Er: YAG laser in soft tissue procedures can be attributed to its 2940 nm wavelength, which perfectly coincides with the maximum absorption peak of water [5,6]. These characteristics enable ‘cold ablation’ in which the target tissue is vaporized precisely, with minimal thermal conduction to surrounding structures [17]. Unlike diode lasers that may cause deep carbonization and subsequent inflammation, Er: YAG laser-mediated excision results in a very thin coagulation layer, which preserves the viability of the adjacent periodontal ligament and periosteum, thereby facilitating faster cellular migration and re-epithelialization [8]. While lasers have increasingly been preferred due to their minimally invasive nature, faster healing, and improved postoperative comfort, there is a paucity of studies directly comparing these approaches with topical agents [7]. By addressing both the surgical technique and adjunctive chemical applications, this study provides novel insights into optimizing patient comfort. wound healing and clinical efficacy following labial frenectomy.

Nowadays, lasers are preferred for frenectomy procedures due to their advantages, including minimal invasiveness, faster healing, and enhanced postoperative comfort [5,12]. Olivi et al. demonstrated that Er: YAG laser–assisted labial frenectomy is a minimally invasive, well-accepted, and predictable technique in children, providing reduced operative time, minimal postoperative discomfort, avoidance of sutures, and stable long-term healing without recurrence [27]. Baxter et al. demonstrated that diode- and CO2–laser–assisted maxillary labial frenectomy in children is a safe and effective procedure that does not hinder orthodontic diastema closure and may facilitate spontaneous reduction in diastema width. with minimal postoperative morbidity [28]. Fonseca et al. conducted a pilot study on diode laser–assisted lower labial frenectomy, reporting that the technique effectively controlled postoperative pain, promoted healing, and minimized bleeding, with most patients showing marked improvement by 30 days postoperatively [17]. In this study, conventional scalpel and Er: YAG laser frenectomy methods were compared across different types of frenulum cases, each treated with Kloroben^®^ or Klorhex Plus^®^.

Numerous studies in the literature have compared different laser modalities with the conventional scalpel technique for performing labial frenectomy, highlighting the clinical efficacy and patient-centered outcomes of these approaches [11,15,16]. Xie et al. reported that Er: YAG and Nd: YAG laser-assisted labial frenectomy provides significantly shorter operative times and reduces postoperative pain, resulting in greater patient comfort compared with the conventional scalpel technique [29]. Uraz et al. reported that 980 nm diode laser–assisted labial frenectomy yields clinical improvements in keratinized and attached gingival tissues comparable to those of the scalpel technique. while significantly reducing postoperative pain. discomfort. and functional complications. making it a safe and patient-comfortable alternative [30]. Calisir and Ege found that Nd: YAG laser–assisted labial frenectomy resulted in lower postoperative pain and improved patient comfort during eating and speaking compared with the conventional technique [31]. Özener et al. reported that diode laser–assisted frenectomy demonstrated clinical outcomes and recurrence rates comparable to those of the conventional technique across different types of abnormal frenulum insertions. supporting the diode laser as an effective alternative [32]. Vincent et al. reported that diode laser (980 nm)–assisted maxillary labial frenectomy provides significantly better clinical outcomes than the conventional scalpel technique, with reduced pain, minimal bleeding, faster wound healing, and improved patient acceptance [16]. Sarmadi et al. reported that Er: YAG laser frenectomy is faster and associated with significantly less intraoperative bleeding than the conventional scalpel technique, while patient satisfaction and wound-healing outcomes were comparable between the two methods [11]. The results of our study indicate that laser-assisted labial frenectomy significantly reduces postoperative pain and analgesic consumption compared with conventional scalpel surgery, particularly during the first postoperative days. Among all protocols, the combination of laser and Klorhex Plus^®^ demonstrated the most pronounced analgesic effect, followed closely by the Laser + Kloroben^®^ group, highlighting the added benefit of laser application in enhancing patient comfort. Landry score analysis further demonstrated that laser-assisted groups achieved faster and higher-quality wound healing, with the Laser Klorhex Plus^®^ group showing the highest proportion of “Excellent” outcomes. These findings collectively reinforce the growing evidence that laser-assisted labial frenectomy. particularly when combined with adjunctive agents such as Klorhex Plus^®^. offers superior postoperative comfort and clinical efficiency compared with conventional scalpel surgery.

Several studies have directly compared different laser modalities for labial frenectomy. evaluating their effects on wound healing, intraoperative bleeding, postoperative pain, and overall patient outcomes [6,7,15]. Onur concluded that both 2780 nm Er.Cr: YSGG and 940 nm diode lasers are safe and effective for pediatric labial frenectomy with low postoperative pain, while the Er.Cr: YSGG laser demonstrated significantly faster wound healing compared to the diode laser [33]. Sobouti et al. reported that maxillary labial frenectomy performed with a diode laser significantly reduced intraoperative bleeding, postoperative pain, and functional discomfort compared with a scalpel, with the 445 nm diode laser demonstrating superior pain control and faster tissue healing than the 980 nm diode laser [34]. Pié-Sánchez et al. reported that both CO_2_ and Er.Cr: YSGG lasers are effective for upper lip frenectomy, with the CO_2_ laser offering better bleeding control and shorter surgical time, and the Er.Cr: YSGG laser providing faster wound healing [35]. Sfasciotti et al. reported that although both diode and CO_2_ lasers are effective for labial frenectomy in pediatric patients, the diode laser demonstrated significantly superior outcomes in several intra- and postoperative parameters, making it the more suitable treatment option [36]. Sayed Taha et al. reported that after upper labial frenectomy, the Er: YAG laser promoted faster wound healing, while the diode laser was more effective in reducing postoperative pain, highlighting complementary advantages of the two laser types [7]. In this study, Er-YAG laser application significantly improved periodontal parameters, reduced postoperative pain and analgesic use, and promoted faster, higher-quality healing. Early hemostasis and epithelialization were also enhanced, highlighting the clinical and patient-centered benefits of Er-YAG laser therapy.

Our results are consistent with existing randomized controlled trials (RCTs) in the literature [37,38]. Like the findings of Sayed Taha et al. [7] and Sarmadi et al. [11], our cohort demonstrated that laser applications yield superior patient-reported outcomes compared to traditional scalpel-based methods. While our study is a retrospective longitudinal cohort, the alignment of our data with high-level RCT evidence reinforces the reliability of using Er: YAG lasers to reduce intraoperative bleeding and postoperative pain [27,34]. However, unlike many RCTs that focus solely on the surgical tool [31,32,34]. Our study adds a new dimension by confirming that the adjunctive use of flurbiprofen-containing mouthwashes further amplifies these benefits. a finding that warrants further investigation in prospective randomized designs.

Although chlorhexidine prescription appears to be the gold standard following frenectomy procedures, a limited number of studies have explored the use of alternative chemical agents to support healing and postoperative outcomes [13,14]. Çankaya et al. found that topical hyaluronic acid application following laser-assisted frenectomy significantly reduced wound surface area and improved patient satisfaction compared to laser treatment alone [39]. Doğan et al. reported that the topical application of 0.6% hyaluronic acid significantly enhanced healing, postoperative comfort, and periodontal outcomes after labial frenectomy in pediatric patients, with the best results observed when combined with diode laser treatment [38]. Priya et al. reported a successful outcome following a conventional scalpel maxillary labial frenectomy, in which the patient was instructed to apply topical ozonated oil three times daily for at least five minutes postoperatively [40]. In this study, Kloroben^®^, which contains both chlorhexidine gluconate, a broad-spectrum antimicrobial agent, and benzydamine hydrochloride, providing additional anti-inflammatory and analgesic effects, and Klorhex Plus^®^, which primarily combines chlorhexidine with flurbiprofen to enhance antimicrobial and anti-inflammatory actions, were used as adjunctive agents following frenectomy procedures. Our findings demonstrate that laser application and Klorhex Plus^®^, both individually and especially in combination, significantly accelerate and enhance postoperative clinical outcomes compared with conventional scalpel treatment. Although all treatment modalities were effective over time, the consistently lowest clinical parameter values observed in the Laser + Klorhex Plus^®^ group indicate a superior synergistic effect in reducing plaque accumulation, gingival inflammation, and bleeding.

In this study, no statistically significant association was found between frenulum type and postoperative healing quality or pain levels following labial frenectomy. Landry scores increased, and VAS scores decreased over time in all frenulum types, indicating a similar pattern of wound healing and pain reduction across groups. These findings suggest that frenulum type alone is not a determining factor for postoperative healing outcomes or patient comfort following labial frenectomy. Although previous studies have mainly associated frenulum type with oral health parameters, diastema presence, and periodontal status [37,41,42], evidence directly evaluating its effect on postoperative healing quality and pain is limited; therefore, the present study provides a valuable contribution by directly addressing the role of frenulum type on surgical outcomes.

From a clinical decision-making perspective, our findings suggest that the choice of surgical modality should extend beyond mere tissue excision. The integration of Er: YAG laser technology, particularly when supplemented with flurbiprofen-containing antimicrobial mouthwashes like Klorhex Plus^®^, offers a significant advantage in managing postoperative morbidity. For practitioners, this synergistic approach not only reduces the immediate need for systemic analgesics but also enhances patient compliance by minimizing early postoperative bleeding and functional discomfort. Therefore, in cases where patient comfort and rapid recovery are prioritized—such as in pediatric or highly anxious patients, the laser-antimicrobial combination should be considered the preferred clinical protocol.

This study has several limitations that should be acknowledged. The retrospective design inherently limited control over data collection, treatment allocation, and protocol standardization, and precluded true randomization, thereby introducing the possibility of selection and allocation bias. Moreover, the single-center design may limit the external validity and generalizability of the findings. Although a post hoc power analysis indicated adequate statistical power, the relatively modest sample size may still limit the robustness of subgroup analyses. All surgical procedures were performed by a single experienced periodontist, which ensured procedural consistency and minimized inter-operator variability; however, operator-related bias cannot be entirely excluded. In addition, differences in age distribution among groups may have acted as a potential confounding factor, despite comparable baseline periodontal parameters. The follow-up period was limited to 28 days, which precludes assessment of long-term outcomes such as tissue stability, scar formation, or recurrence of abnormal frenulum insertions. Furthermore, several secondary outcomes, including postoperative pain, analgesic consumption, bleeding, and adherence to postoperative oral hygiene and mouthwash protocols, were based on patient self-reporting and are therefore subject to recall and reporting bias. Additionally, while a standardized 7-day mouthwash protocol was prescribed to all groups, the absence of sutures in the laser-assisted groups may have influenced patient adherence compared to the conventional groups, where suture removal on the 7th day provided a definitive clinical endpoint. This potential for differential exposure is a limitation inherent to comparing sutured and non-sutured surgical techniques. Finally, although different antimicrobial agents were evaluated in combination with conventional and laser-assisted frenectomy, the study design did not allow for complete isolation of the individual effects of surgical technique and adjunctive chemical therapy. Future well-designed prospective, randomized, multicenter trials with larger sample sizes, longer follow-up periods, and standardized laser parameters are warranted to validate and expand upon the present findings.

## 5. Conclusions

In conclusion, within the limitations of this retrospective cohort study, the use of the Er: YAG laser for labial frenectomy was linked to lower postoperative pain and improved wound-healing outcomes compared with the conventional technique. Furthermore, the adjunctive use of a flurbiprofen-containing antimicrobial mouthwash (Klorhex Plus^®^) was linked to favorable clinical and patient-reported outcomes. These findings suggest a positive association between the combined laser-antimicrobial approach and improved postoperative recovery, although prospective randomized trials are necessary to establish a definitive causal relationship.

## Figures and Tables

**Table 1:** General descriptive statistics of patients’ demographic/clinical parameters.

		Group 1 (*n* = 27)		Group 2 (*n* = 24)		Group 3 (*n* = 25)		Group 4 (*n* = 26)		Total (*n* = 102)	
		N	%	N	%	N	%	N	%	N	%
Gender	Male	13	48.1%	11	45.8%	12	48.0%	13	50.0%	49	48.0%
	Female	14	51.9%	13	54.2%	13	52.0%	13	50.0%	53	52.0%
Age	18–24	9	33.3%	2	8.3%	12	48.0%	14	53.8%	37	36.3%
	25–44	10	37.0%	12	50.0%	4	16.0%	5	19.2%	31	30.4%
	45–60	8	29.6%	10	41.7%	9	36.0%	7	26.9%	34	33.3%
Number of brushings per day	1	4	14.8%	3	12.5%	7	28.0%	4	15.4%	18	17.6%
	2	23	85.2%	21	87.5%	18	72.0%	22	84.6%	84	82.4%
Smoking	No	22	81.5%	17	70.8%	21	84.0%	21	80.8%	81	79.4%
	Yes	5	18.5%	7	29.2%	4	16.0%	5	19.2%	21	20.6%
Systemic Disease	Yes	1	3.7%	0	0.0%	3	12.0%	5	19.2%	9	8.8%
	No	26	96.3%	24	100.0%	22	88.0%	21	80.8%	93	91.2%
Frenulum Type	Gingival	9	33.3%	5	20.8%	4	16.0%	6	23.1%	24	23.5%
	Mucosal	4	14.8%	4	16.7%	3	12.0%	4	15.4%	15	14.7%
	Papillary Penetrating	4	14.8%	4	16.7%	4	16.0%	4	15.4%	16	15.7%
	Papillary	10	37.0%	11	45.8%	14	56.0%	12	46.2%	47	46.1%

**Table 2:** Comparison results of periodontal clinical parameters by groups and time.

	Group 1 (*n* = 27)		Group 2 (*n* = 24)		Group 3 (*n* = 25)		Group 4 (*n* = 26)		
	Mean	Std. Dev.	Mean	Std. Dev.	Mean	Std. Dev.	Mean	Std. Dev.	* p.
PI Baseline (T0)	0.54	0.15	0.52	0.15	0.51	0.15	0.50	0.15	*0.816*
PI 7th day (T1)	1.29 a	0.17	0.88 c	0.16	1.00 b	0.17	0.75 d	0.15	***0.001***
PI 14th day (T2)	0.85 a	0.11	0.60 b	0.11	0.62 b	0.11	0.51 c	0.11	***0.001***
PI 28th day (T3)	0.53 a	0.06	0.35 b	0.06	0.38 b	0.06	0.29 c	0.05	***0.001***
** p.	***0.001***		***0.001***		***0.001***		***0.001***		
PD Baseline (T0)	2.70	0.47	2.71	0.46	2.68	0.48	2.65	0.49	*0.975*
PD 7th day (T1)	3.00 a	0.00	2.71 b	0.46	3.00 a	0.00	2.23 c	0.43	***0.001***
PD 14th day (T2)	2.63 a	0.49	2.25 b	0.44	2.32 b	0.48	2.00 c	0.00	***0.001***
PD 28th day (T3)	2.37 a	0.49	2.00 b	0.00	2.00 b	0.00	2.00 b	0.00	***0.001***
** p.	***0.001***		***0.001***		***0.001***		***0.001***		
GI Baseline (T0)	1.36	0.08	1.35	0.08	1.35	0.08	1.35	0.08	*0.995*
GI 7th day (T1)	1.61 a	0.06	1.25 c	0.04	1.40 b	0.06	1.05 d	0.04	***0.001***
GI 14th day (T2)	0.97 a	0.06	0.74 b	0.03	0.76 b	0.03	0.55 c	0.04	***0.001***
GI 28th day (T3)	0.65 a	0.03	0.49 c	0.03	0.51 b	0.03	0.36 d	0.03	***0.001***
** p.	***0.001***		***0.001***		***0.001***		***0.001***		
PBI Baseline (T0)	1.36	0.07	1.36	0.07	1.36	0.07	1.36	0.06	*0.997*
PBI 7th day (T1)	1.71 a	0.07	1.37 b	0.04	1.40 b	0.06	1.06 c	0.11	***0.001***
PBI 14th day (T2)	1.01 a	0.03	0.78 b	0.13	0.75 b	0.04	0.54 c	0.10	***0.001***
PBI 28th day (T3)	0.66 a	0.03	0.62 a	0.13	0.54 b	0.09	0.37 c	0.10	***0.001***

* One-way ANOVA test. a, b, c, d: Indicates differences between groups at the same time (Duncan post hoc test). ⟶ ** Repeated ANOVA test ↓.

**Table 3:** Relationship and distribution between groups and Landry (WHI) scores.

		Group 1 (*n* = 27)		Group 2 (*n* = 24)		Group 3 (*n* = 25)		Group 4 (*n* = 26)		
		N	%	N	%	N	%	N	%	* p.
Landry 7th day	Very Poor	0	0.0%	0	0.0%	0	0.0%	0	0.0%	*0.001*
	Poor	4	66.7%	0	0.0%	2	33.3%	0	0.0%	
	Good	15	38.5%	4	10.3%	14	35.9%	6	15.4%	
	Very Good	8	14.0%	20	35.1%	9	15.8%	20	35.1%	
	Excellent	0	0.0%	0	0.0%	0	0.0%	0	0.0%	
Landry 14th day	Very Poor	0	0.0%	0	0.0%	0	0.0%	0	0.0%	*0.001*
	Poor	0	0.0%	0	0.0%	0	0.0%	0	0.0%	
	Good	3	50.0%	0	0.0%	3	50.0%	0	0.0%	
	Very Good	19	35.2%	11	20.4%	16	29.6%	8	14.8%	
	Excellent	5	11.9%	13	31.0%	6	14.3%	18	42.9%	
Landry 28th day	Very Poor	0	0.0%	0	0.0%	0	0.0%	0	0.0%	*0.001*
	Poor	0	0.0%	0	0.0%	0	0.0%	0	0.0%	
	Good	0	0.0%	0	0.0%	0	0.0%	0	0.0%	
	Very Good	8	72.7%	0	0.0%	3	27.3%	0	0.0%	
	Excellent	19	20.9%	24	26.4%	22	24.2%	26	28.6%	

* Significance level according to chi-square or Fisher’s Exact test results. The robustness of the intergroup differences was confirmed by non-parametric cross-validation (*p* < 0.05).

**Table 4:** Relationships and distribution between groups and complete epithelialization (CE) (H_2_O_2_ bubbling).

		Group 1 (*n* = 27)		Group 2 (*n* = 24)		Group 3 (*n* = 25)		Group 4 (*n* = 26)		
		N	%	N	%	N	%	N	%	* p.
CE 7th day (H_2_O_2_ bubbling)	No	0	0.0%	14	35.9%	9	23.1%	16	41.0%	***0.001***
	Yes	27	42.9%	10	15.9%	16	25.4%	10	15.9%	
CE 14th day (H_2_O_2_ bubbling)	No	21	24.1%	24	27.6%	19	21.8%	23	26.4%	*0.063*
	Yes	6	40.0%	0	0.0%	6	40.0%	3	20.0%	
CE 28th day (H_2_O_2_ bubbling)	No	26	26.0%	24	24.0%	24	24.0%	26	26.0%	*0.579*
	Yes	1	50.0%	0	0.0%	1	50.0%	0	0.0%	

* Significance level according to chi-square or Fisher’s Exact test results.

**Table 5:** Multivariable regression analysis of wound healing.

Criteria: Wound Healing	B	SE	*p.*	Odds Ratio	95% CI for Odds	
					Lower	Upper
Age	−0.034	0.028	*0.236*	0.967	0.915	1.022
Smoking (Yes)	−0.068	0.929	*0.942*	0.935	0.151	5.770
Gender (Male)	0.254	0.750	*0.735*	1.289	0.296	5.607
Systemic Disease (Yes)	−0.057	0.125	*0.691*	0.602	0.214	1.521
* Model Summary:	−2 Log Likelihood		Cox & Snell R^2^		Nagelkerke R^2^	
	59.36		0.015		0.033	

* Logistic Regression analysis results. Method: Enter. Dependent Variable: Wound Healing.

**Table 6:** Comparison of VAS scores and number of analgesics by group and time.

	Group 1 (*n* = 27)		Group 2 (*n* = 24)		Group 3 (*n* = 25)		Group 4 (*n* = 26)		
	Mean	Std. Dev.	Mean	Std. Dev.	Mean	Std. Dev.	Mean	Std. Dev.	* p.
VAS 2nd hour	A 4.19	2.50	A 3.04	1.40	A 3.36	2.94	A 2.92	1.20	*0.141*
VAS 1st day	B 2.89 a	2.12	B 1.79 b	1.25	B 2.68 a	2.72	B 1.65 b	1.02	***0.047***
VAS 2nd day	B 2.04	2.41	B 1.13	1.15	C 1.60	1.85	B 1.15	0.78	*0.172*
VAS 3rd day	C 1.30	1.92	C 0.50	0.78	C 1.20	1.38	C 0.69	0.62	*0.088*
VAS 4th day	C 1.00	1.59	C 0.37	0.58	D 0.64	0.81	C 0.35	0.49	*0.065*
VAS 5th day	C 0.74 a	1.06	C 0.33 c	0.48	D 0.52 b	0.59	C 0.12 c	0.33	***0.009***
VAS 6th day	D 0.44 a	0.58	C 0.29 b	0.46	D 0.44 a	0.51	C 0.08 c	0.27	***0.018***
VAS 7th day	D 0.33 a	0.48	C 0.17 b	0.38	D 0.16 b	0.37	C 0.04 c	0.20	***0.044***
** p.	***0.001***		***0.001***		***0.001***		***0.001***		
Analgesic 2nd hour	A 1.22 a	0.89	A 0.54 b	0.51	A 0.52 b	0.59	A 0.54 b	0.51	***0.001***
Analgesic 1st day	B 0.74 a	0.86	B 0.21 c	0.41	B 0.28 c	0.46	A 0.42 b	0.50	***0.009***
Analgesic 2nd day	B 0.48	0.94	B 0.21	0.41	B 0.28	0.54	B 0.04	0.20	*0.063*
Analgesic 3rd day	B 0.22	0.64	B 0.21	0.41	C 0.16	0.37	C 0.00	0.00	*0.228*
Analgesic 4th day	B 0.19	0.62	B 0.17	0.38	C 0.16	0.37	C 0.00	0.00	*0.344*
Analgesic 5th day	C 0.04	0.19	C 0.04	0.20	D 0.00	0.00	C 0.00	0.00	*0.572*
Analgesic 6th day	C 0.04	0.19	C 0.04	0.20	D 0.00	0.00	C 0.00	0.00	*0.572*
Analgesic 7th day	D 0.00	0.00	D 0.00	0.00	D 0.00	0.00	C 0.00	0.00	.
** p.	***0.001***		***0.001***		***0.001***		***0.001***		

* One-way ANOVA test. a.b.c.: Indicates differences between groups at the same time (Duncan post hoc test) ⟶ ** Repeated ANOVA test. A.B.C.D.: Indicates differences between times within the same group (Bonferroni Post Hoc test). The robustness of the intergroup differences was confirmed by non-parametric cross-validation (*p* < 0.05) ↓.

**Table 7:** Relationships and distribution between groups, and the presence of bleeding.

		Group 1 (*n* = 27)		Group 2 (*n* = 24)		Group 3 (*n* = 25)		Group 4 (*n* = 26)		
		N	%	N	%	N	%	N	%	* p.
Bleeding 2nd hour	No	11	17.2%	20	31.3%	13	20.3%	20	31.3%	***0.004***
	Yes	16	42.1%	4	10.5%	12	31.6%	6	15.8%	
Bleeding 1st day	No	18	23.7%	20	26.3%	17	22.4%	21	27.6%	*0.399*
	Yes	9	34.6%	4	15.4%	8	30.8%	5	19.2%	
Bleeding 2nd day	No	22	24.2%	24	26.4%	20	22.0%	25	27.5%	***0.044***
	Yes	5	45.5%	0	0.0%	5	45.5%	1	9.1%	
Bleeding 3rd day	No	24	24.7%	24	24.7%	23	23.7%	26	26.8%	*0.149*
	Yes	3	60.0%	0	0.0%	2	40.0%	0	0.0%	
Bleeding 4th day	No	26	26.0%	24	24.0%	24	24.0%	26	26.0%	*0.579*
	Yes	1	50.0%	0	0.0%	1	50.0%	0	0.0%	
Bleeding 5th day	No	27	26.5%	24	23.5%	25	24.5%	26	25.5%	.
	Yes	0	0.0%	0	0.0%	0	0.0%	0	0.0%	
Bleeding 6th day	No	27	26.5%	24	23.5%	25	24.5%	26	25.5%	.
	Yes	0	0.0%	0	0.0%	0	0.0%	0	0.0%	
Bleeding 7th day	No	27	26.5%	24	23.5%	25	24.5%	26	25.5%	.
	Yes	0	0.0%	0	0.0%	0	0.0%	0	0.0%	

* Significance level according to chi-square or Fisher’s Exact test results.

## Data Availability

The datasets used and/or analyzed during the current study are available from the corresponding author on reasonable request.

## Supplementary Materials

The following supporting information can be downloaded at https://www.mdpi.com/article/10.3390/jcm15062419/s1: Supplementary Table S1: Relationship and distribution between groups and patient demographic and clinical information; Supplementary Table S2: The relationship and distribution between frenulum type and Landry score; Supplementary Table S3: Comparison results of VAS scores according to frenulum type; STROBE checklist.
