# Supplementary material for: Impact of Antimicrobial Mouthwash on Outcomes of Er: YAG Laser Versus Scalpel Frenectomy: A Retrospective Longitudinal Cohort Study
Source: J Clin Med. 2026 Mar 21;15(6):2419. doi: 10.3390/jcm15062419 (PMC13026365; doi:10.3390/jcm15062419)
Supplement: Supplementary file 1 [file jcm-15-02419-s001.zip › jcm-4186295-supplementary tables.pdf]

Supplementary Table S1. Relationship and distribution between groups and patient demographic and clinical information

		Group 1 (*n*=27)		Group 2 (*n*=24)		Group 3 (*n*=25)		Group 4 (*n*=26)		
		N	%	N	%	N	%	N	%	***p.**
Gender	Male	13	26.5%	11	22.4%	12	24.5%	13	26.5%	*.993*
	Female	14	26.4%	13	24.5%	13	24.5%	13	24.5%	
Age	18-24	9	24.3%	2	5.4%	12	32.4%	14	37.8%	***.024***
	25-44	13	31.0%	15	35.7%	7	16.7%	7	16.7%	
	45-60	5	21.7%	7	30.4%	6	26.1%	5	21.7%	
Number of brushings per day	1	4	22.2%	3	16.7%	7	38.9%	4	22.2%	*.471*
	2	23	27.4%	21	25.0%	18	21.4%	22	26.2%	
Smoking	No	22	27.2%	17	21.0%	21	25.9%	21	25.9%	*.682*
	Yes	5	23.8%	7	33.3%	4	19.0%	5	23.8%	
Systemic Disease	Yes	1	11.1%	0	0.0%	3	33.3%	5	55.6%	*.071*
	No	26	28.0%	24	25.8%	22	23.7%	21	22.6%	
Frenulum Type	Gingival	9	37.5%	5	20.8%	4	16.7%	6	25.0%	*.963*
	Mucosal	4	26.7%	4	26.7%	3	20.0%	4	26.7%	
	Papillary Penetrating	4	25.0%	4	25.0%	4	25.0%	4	25.0%	
	Papillary	10	21.3%	11	23.4%	14	29.8%	12	25.5%	

* Significance level according to chi-square or Fisher's Exact test results

Supplementary Table S2. The relationship and distribution between frenulum type and Landry score

		Frenulum Type								
		Gingival		Mucosal		Papillary Penetrating		Papillary		
		N	%	N	%	N	%	N	%	**p.*
Landry 7th day	Very Poor	0	0.0%	0	0.0%	0	0.0%	0	0.0%	*.349*
	Poor	2	8.3%	0	0.0%	0	0.0%	4	8.5%	
	Good	7	29.2%	4	26.7%	9	56.3%	19	40.4%	
	Very Good	15	62.5%	11	73.3%	7	43.8%	24	51.1%	
	Excellent	0	0.0%	0	0.0%	0	0.0%	0	0.0%	
Landry 14th day	Very Poor	0	0.0%	0	0.0%	0	0.0%	0	0.0%	*.818*
	Poor	0	0.0%	0	0.0%	0	0.0%	0	0.0%	
	Good	2	8.3%	0	0.0%	0	0.0%	4	8.5%	
	Very Good	12	50.0%	8	53.3%	9	56.3%	25	53.2%	
	Excellent	10	41.7%	7	46.7%	7	43.8%	18	38.3%	
Landry 28th day	Very Poor	0	0.0%	0	0.0%	0	0.0%	0	0.0%	*.126*
	Poor	0	0.0%	0	0.0%	0	0.0%	0	0.0%	
	Good	0	0.0%	0	0.0%	0	0.0%	0	0.0%	
	Very Good	3	12.5%	0	0.0%	0	0.0%	8	17.0%	
	Excellent	21	87.5%	15	100.0%	16	100.0%	39	83.0%	

* Significance level according to chi-square or Fisher's Exact test results

Supplementary Table S3. Comparison results of VAS scores according to frenulum type

	Frenulum Type								
	Gingival		Mucosal		Papillary Penetrating		Papillary		
	Mean	Std. Dev.	Mean	Std. Dev.	Mean	Std. Dev.	Mean	Std. Dev.	**p.*
VAS 2nd hour	A 2.71	1.94	A 2.87	2.17	A 3.25	1,81	A 3,96	2,30	*,087*
VAS 1st day	B 1.63	1.79	A 2.87	2.39	B 2.00	1,97	B 2,49	1,84	*,179*
VAS 2nd day	C .92	1.44	B 1.87	1.51	C 1.44	1,63	C 1,68	1,88	*,258*
VAS 3rd day	D .58	1.25	B 1.27	1.58	C 1.00	1,21	D ,98	1,31	*,440*
VAS 4th day	D .42	1.06	C .67	.98	D .75	1,00	D ,62	1,01	*,752*
VAS 5th day	D .29	.86	C .53	.74	D .50	,63	E ,45	,65	*,710*
VAS 6th day	D .17	.38	C .33	.49	D .38	,50	E ,36	,53	*,409*
VAS 7th day	D .17	.38	D .27	.46	E .06	,25	E ,19	,40	*,512*
***p.*	***.001***		***.001***		***.001***		***.001***		

* One-way ANOVA test. a.b.c.: Indicates differences between groups at the same time (Duncan post-hoc test) → ** Repeated ANOVA test. A.B.C.: Indicates differences between times within the same group (Bonferroni Post-Hoc test) ↓
